# The prevalence of ADH1B and OPRM1 alleles predisposing for alcohol consumption are increased in the Hungarian psoriasis population

**DOI:** 10.1007/s00403-019-01915-y

**Published:** 2019-04-22

**Authors:** Zita Szentkereszty-Kovács, Szilvia Fiatal, Andrea Szegedi, Dóra Kovács, Eszter Janka, Krisztina Herszényi, Péter Holló, Pernilla Nikamo, Mona Ståhle, Éva Remenyik, Dániel Törőcsik

**Affiliations:** 1 Department of Dermatology, Faculty of Medicine, University of Debrecen, Debrecen, Hungary; 2 Department of Preventive Medicine, Faculty of Public Health, University of Debrecen, Debrecen, Hungary; 3 Division of Dermatological Allergology, Faculty of Medicine, University of Debrecen, Debrecen, Hungary; 4 Department of Dermatology, Venereology and Dermatooncology, Semmelweis University, Budapest, Hungary; 5 Unit of Dermatology and Venereology, Department of Medicine, Karolinska lnstitutet, Karolinska University Hospital, Stockholm, Sweden

**Keywords:** Psoriasis, Alcohol consumption, ADH1B gene, OPRM1 gene, Population genetics

## Abstract

Alcohol intake affects in great the symptoms and life of  psoriasis patients, although the association of SNPs related to increased alcohol consumption with psoriasis has not been elucidated. Therefore, to investigate the association of psoriasis with established alcohol consumption and dependence-related gene variants we conducted a population-based case–control study including 3743 subjects (776 psoriasis cases and 2967 controls from the general Hungarian population). Genotyping of 23 SNPs at *ADH1B, ADH1C, ALDH1A1, ALDH2, SLC6A3, DDC, GABRA2, GABRG1, HTR1B, MAOA, TPH2, CHRM2, GRIN2A, POMC, OPRM1, OPRK1* and *BDNF* were determined and differences in genotype and allele distributions were investigated. Multiple logistic regression analyses were implemented. Analysis revealed association between C allele of the rs1229984 polymorphism (*ADH1B* gene) and psoriasis risk (OR_additive_ = 1.58, 95% CI 1.23–2.03, *p* < 0.001, OR_recessive_ = 1.58, 95% CI 1.22–2.04, *p* = 0.001). Furthermore, the G allele of rs1799971 polymorphism (*OPRM1* gene) increased the risk of familial aggregation (OR_additive_ = 1.99, 95% CI 1.36–2.91, *p* < 0.001 OR_dominant_ = 2.01, 95% CI 1.35–3.01, *p* < 0.001). In subgroups of psoriatic patients with history of early onset and familial aggregation effect allele ‘C’ of rs1229984 showed association in the additive and recessive models (OR_additive_ = 2.41, 95% CI 1.26–4.61, *p* < 0.01, OR_recessive_ = 2.42, 95% CI 1.26–4.68, *p* < 0.01). While effect allele ‘G’ of rs1799971 (*OPRM1*) also associated with increased risk of early onset and familial aggregation of psoriasis in the additive and dominant models (OR_additive_ = 1.75, 95% CI 1.27–2.43, *p* = 0.001, OR_dominant_ = 1.82, 95% CI 1.26–2.63, *p* = 0.001). Our results suggest that genetically defined high-risk individuals for alcohol consumption are more common in the psoriasis population.

**Electronic supplementary material:**

The online version of this article (10.1007/s00403-019-01915-y) contains supplementary material, which is available to authorized users.

## Introduction

Psoriasis is a common chronic inflammatory disease affecting 1–4% of the world population, with strong genetic as well as environmental factors in the background [32] affecting the onset, clinical manifestation and the course of the disease [6]. While the genetic association with the HLA-Cw6 allele and the nearly 40 risk regions revealed by recent GWAS studies confirm a pivotal role for the immune system [31, 33], the extent and mechanisms for gene-environment interactions in contributing to the pathogenesis or the increased risk for various comorbidities remains to be elucidated.

Alcohol consumption is one of the primary environmental factors, showing a direct link with psoriatic risk [3, 11, 20, 37, 49, 50], although further studies are needed to confirm if excessive alcohol consumption correlates with disease severity [17, 27, 36, 51]. Alcohol intake may affect psoriasis at various levels from the lower compliance of alcoholic patients to its interaction with the metabolism of therapeutic drugs [18]. Moreover, alcohol was shown to have a direct effect on immune cells and keratinocytes promoting their psoriasis related phenotype [12–14]. Still, at least to our knowledge, no studies were carried out to investigate the possible genetic background for alcohol consumption in psoriasis populations, to explore whether psoriatic patients have a genetically increased risk for alcohol consumption.

Heritability in alcohol dependence is independent of gender and is estimated to range from 40 to 70% based on twin and adoption studies [10, 48]. Most of the genes and their polymorphisms that have been linked to alcohol dependence both in a predisposing as well as a protective way are related to alcohol metabolism and neurotransmission [7, 46].

In this study, the genetic susceptibility to harmful alcohol consumption in Hungarian psoriasis patients was compared to subjects of general population, by analyzing and comparing the frequencies of 23 alleles associated with alcohol consumption behaviors based on literature review and recent publication [7]. We aimed to address if genetic factors driving alcohol consumption could be linked to psoriasis, or whether primarily environmental factors contribute to the increased alcohol consumption of psoriatic patients [9].

## Materials and methods

### Study population and characteristics

A total of 776 patients (580 patients from Debrecen region and 196 patients from Budapest region) diagnosed with psoriasis vulgaris and 2967 individuals from the Hungarian general population (HG) were enrolled in the study.

The diagnosis of psoriasis was approved by at least two dermatologists. Data including family history (familial aggregation was positive if psoriasis existed in at least one more case among first degree relatives extended with grandparents in familial anamnestic data) and age at onset (early onset: ≤ 40 years, late onset > 40 years) were collected and their association with HLA-Cw*0602 allele was defined. To assess the severity of psoriasis Psoriasis Area and Severity Index (PASI) score was used. Symptoms were dichotomized as follows: in case of patients receiving only topical treatment and/or PASI < 10 without therapy was specified as mild psoriasis, ≥ 10 as severe psoriasis. Patients receiving systemic therapy were all included in the severe psoriasis group independent of the recent PASI score.

Sample representative of the HG population in terms of geographic, age and sex distributions were obtained from a population-based disease registry, the General Practitioners’ Morbidity Sentinel Stations Programme (GPMSSP) [43]. Our study involved HG subjects investigated during a recent cross-sectional survey. Details of sampling methodology and data collection are described elsewhere [44].

Informed consent was obtained from all individual participants included in the study. The study protocol was approved by the Regional Institutional Scientific and Research Ethical Board of the University of Debrecen and by the Semmelweis University, Budapest, Hungary in accordance with the principles from the Declaration of Helsinki.

### SNP selection

A recent study conducted systematic literature review (PubMed) to identify SNPs of candidate genes most likely to be associated with the characteristics of alcohol consumption by encoding enzymes involved in alcohol metabolism and in pathways related to dependence [7]. Our study utilized the same collection set: alcohol dehydrogenase (*ADH1B*, *ADH1C*), aldehyde dehydrogenase (*ALDH1A1, ALDH2*) and neurotransmitters in the dopaminergic (*SLC6A3, DDC*), GABAergic (*GABRA2, GABRG1*), serotonergic (*HTR1B, MAOA, TPH2*), cholinergic (*CHRM2*), glutamatergic (*GRIN2A*) and opioidergic (*POMC, OPRM1, OPRK1*) pathways, as well as one SNP in the gene encoding a protein involved in neural development and dendritic growth neurogenesis (*BDNF*) (Supplementary Table 1).

### DNA preparation

DNA isolation was performed from ethylenediaminetetraacetic acid-anticoagulated blood samples using the MagNA Pure LC DNA Isolation Kit—Large Volume (Roche Diagnostics, Mannheim, Germany) according to the manufacturer’s protocol. The extracted DNA samples were eluted in MagNA Pure LC DNA Isolation Kit-Large Volume Elution Buffer (Roche Diagnostics) and stored in − 30 °C until measurements.

### Genotype assessment

Genotyping of the selected 23 SNPs was performed by Mutation Analysis Core Facility (MAF) of Clinical Research Center, Karolinska University Hospital (Stockholm, Sweden) using the Mass Array platform with iPLEX Gold Chemistry (Sequenom). The validation, concordance analysis and quality control were conducted by MAF according to their protocol, resulting in a successful genotyping outcome for 3433 (776 psoriatic and 2917 HG) DNA samples. HLA-Cw*0602 typing was carried out as described previously [34].

### Statistical analyses

The data were analyzed using STATA 12.0 Statistical software (StataCorp LP, College Station, TX, USA). The Mann–Whitney *U* and *χ*^2^ tests were used to compare the mean age and sex distribution of the two study groups. The existence of Hardy–Weinberg equilibrium (HWE) and significant differences in the allele and genotype frequencies between the two populations were examined with the *χ*^2^ test. To decrease the proportion of false positive results the *p* threshold of 0.002 was applied (Bonferroni correction); otherwise the threshold for significance was 0.05.

To take account of confounding effects of gender and age on differences between study populations, linear regression models were constructed. Psoriatic samples were divided into several subgroups defined by the clinical parameters such as familial aggregation, age at onset, and severity as described previously. Association analyses were done according to additive, dominant and recessive models. To further confirm the findings two psoriatic populations from Hungary (Budapest vs. Debrecen) were also compared.

To assess whether any allele frequency differences exist between the Debrecen and the Budapest subgroups of the patients *χ*^2^ tests were performed. Similar genotype distribution of the SNPs were found in these subgroups, suggesting that the association of psoriasis with the identified risk alleles in the Hungarian psoriatic patients, is independent of the regional origin of the patients.

To assess gene–gene interactions between HLA-Cw*0602 and the ADH1B and OPRM1 genes PLINK software epistasis command was used [38].

## Results

### Comparison of psoriasis population to Hungarian general population

The mean age was 49.15 years ± 16.82 in the case of psoriatic patients and 45.53 years ± 14.62 in the case of the HG population. The mean age of the study groups was significantly different according to the Mann–Whitney *U* test (*p* < 0.001). The proportion of male individuals in psoriatic sample was significantly higher (psoriatic: 60.6% vs. HG: 46.8%, *p* < 0.001).

Out of the 23 SNPs investigated, the genotype distribution of rs1386496 (*TPH2* gene) deviated from Hardy–Weinberg equilibrium in the HG group (*p* < 0.001) and was thus excluded from further analysis. Allele frequencies in the study populations, which were calculated on the basis of the obtained genotype distributions, are shown in Table 1. *ALDH2* rs671 and *SLC6A3* rs6530 were monomorphic in both groups, and were consequently excluded from further analyses.

**Table 1:** Effect allele frequency distribution of the investigated SNPs

Gene	SNP	Effect allele	Effect allele frequency among psoriasis patients, *N* = 776	Effect allele frequency in general population, *N* = 2967	*p*	Model	OR (95% CI)	*p*
POMCs	rs1866146	G	0.3807	0.3955	0.299	ADD	0.94 (0.83–1.06)	0.3295
						DOM	0.85 (0.72–1.01)	0.059
						REC	1.08 (0.86–1.36)	0.505
POMC	rs6713532	C	0.2627	0.2619	0.951	ADD	0.999 (0.87–1.14)	0.9885
						DOM	1.06 (0.89–1.25)	0.518
						REC	0.79 (0.55–1.12)	0.185
GABRG1	rs2221020	C	0.5183	0.5176	0.958	ADD	0.99 (0.89–1.12)	0.9233
						DOM	0.94 (0.78–1.14)	0.525
						REC	1.04 (0.87–1.25)	0.652
GABRA2	rs567926	G	0.3997	0.3954	0.762	ADD	1.01 (0.90–1.14)	0.8607
						DOM	1.05 (0.88–1.25)	0.5801
						REC	0.96 (0.76–1.2)	0.694
GABRA2	rs279871	T	0.6112	0.6126	0.92	ADD	0.99 (0.88–1.12)	0.9327
						DOM	1.13 (0.89–1.43)	0.3079
						REC	0.93 (0.78–1.10)	0.3799
GABRA2	rs279858	C	0.3914	0.3869	0.751	ADD	1.02 (0.90–1.15)	0.7629
						DOM	1.11 (0.93–1.31)	0.2532
						REC	0.89 (0.71–1.13)	0.3399
ADH5	rs1154400	C	0.3377	0.3405	0.843	ADD	0.97 (0.86–1.10)	0.6861
						DOM	0.98 (0.83–1.17)	0.8557
						REC	0.93 (0.71–1.21)	0.573
ADH4	rs7694646	A	0.3191	0.3043	0.272	ADD	1.07 (0.94–1.21)	0.3234
						DOM	1.06 (0.90–1.25)	0.5137
						REC	1.06 (0.89–1.53)	0.278
ADH4	rs1800759	G	0.5885	0.5932	0.746	ADD	0.9991(0.89–1.13)	0.9888
						DOM	0.86 (0.69–1.07)	0.1691
						REC	1.098 (0.92–1.30)	0.2906
ADH1B	rs1229984	C	0.946	0.9204	0.001	ADD	1.58 (1.23–2.03)	0.000371
						DOM	4.29 (0.55–33.08)	0.163
						REC	1.58 (1.22–2.04)	0.001
ADH7	rs1154458	C	0.4207	0.412	0.543	ADD	1.06 (0.95–1.20)	0.3015
						DOM	1.05 (0.88–1.25)	0.5672
						REC	1.14 (0.92–1.41)	0.2906
SLC6A3	rs463379	G	0.7849	0.7746	0.396	ADD	1.05 (0.91–1.21)	0.5035
						DOM	1.56 (1.01–2.40)	0.04589
						REC	0.99 (0.84–1.18)	0.9367
HTR1B	rs130058	T	0.7167	0.7234	0.608	ADD	0.96 (0.85–1.10)	0.582
						DOM	1.08 (0.79–1.47)	0.631
						REC	0.92 (0.78–1.09)	0.3355
OPRM1	rs1799971	G	0.132	0.1311	0.927	ADD	0.99 (0.83–1.18)	0.8785
						DOM	1.01 (0.83–1.22)	0.9315
						REC	0.69 (0.31–1.51)	0.3493
DDC	rs3779084	G	0.21	0.2019	0.487	ADD	1.06 (0.93–1.21)	0.37
						DOM	1.05 (0.88–1.25)	0.6003
						REC	1.2 (0.89–1.60)	0.2352
CHRM2	rs324650	A	0.5026	0.5117	0.531	ADD	0.96 (0.85–1.08)	0.4842
						DOM	0.94 (0.78–1.14)	0.5233
						REC	0.95 (0.79–1.15)	0.6137
OPRK1	rs6985606	T	0.4735	0.4734	0.994	ADD	1.004 (0.89–1.13)	0.9522
						DOM	1.01 (0.84–1.22)	0.8812
						REC	0.99 (0.81–1.21)	0.9533
ALDH1A1	rs610529	G	0.4397	0.4467	0.63	ADD	0.98 0.87–1.10)	0.7383
						DOM	1.05 (0.88–1.26)	0.6057
						REC	0.88 (0.71–1.09)	0.2373
BDNF	rs6265	C	0.8197	0.8003	0.093	ADD	1.15 (0.98–1.33)	0.08108
						DOM	1.27 (0.79–2.05)	0.3276
						REC	1.16 (0.97–1.38)	0.09954
GRIN2A	rs2072450	C	0.8687	0.8641	0.646	ADD	1.17 (0.90–1.27)	0.443
						DOM	1.3 (0.69–2.42)	0.4179
						REC	1.06 (0.87–1.29)	0.549
MAOA	rs979606	T	0.6849	0.6832	0.916	ADD	1.013 (0.873– 1.176)	0.862
						DOM	NA^a^	NA^a^
						REC	NA^a^	NA^a^

Multiple test correction revealed significant differences for rs1229984 *(ADH1B)* between the psoriatic and HG population

^a^*NA* not applicable, rs979606 was X-linked

Differences between the psoriatic and HG population remained significant only for one SNP (rs1229984) after multiple test correction. Comparing the allele frequency distribution of SNP rs1229984 in the gene coding alcohol dehydrogenase 1B (*ADH1B*) significantly higher prevalence of effect allele C was found in the psoriatic population compared to the HG population (94.46% vs. 92.04%, *p* < 0.001, respectively). Significant differences were observed between study groups in the association analysis according to the additive (OR_additive_ = 1.58, 95% CI 1.23–2.03, *p* < 0.001) and the recessive model (OR_recessive_ = 1.58, 95% CI 1.22–2.04, *p* = 0.001) but not the dominant model (OR_dominant_ = 4.29, 95% CI 0.55–33.08, *p* = 0.163) (Table 1).

### Stratified analysis of psoriasis group

By comparing the psoriasis subgroups several strata of psoriatic patients were defined, such as familial aggregation vs. sporadic case, early-onset of disease (≤ 40 years) vs. late-onset (> 40 years) and mild (PASI score < 10) vs. severe psoriasis symptoms (PASI score ≥ 10 or receiving systemic therapy). The number of psoriatic subjects in the subgroup analysis (Supplementary Table 2) were sufficient to attain power of 80% and to detect an OR of 1.9 (assuming at least case–control ratio of 1.8 and alfa = 0.05). The statistical power was calculated using Epiinfo 7.2 StatCalc calculator.

Significant results were found only in case of one SNP, rs1799971 (*μ*-opioid receptor gene, *OPRM1*) when familial cases were compared to sporadic cases, The effect allele G increased the risk of familial psoriasis by twofold compared to sporadic cases both in additive and in dominant models (OR_additive_ = 1.99, 95% CI 1.36–2.91, *p* < 0.001, OR_dominant_ = 2.01, 95% CI 1.35–3.01, *p* < 0.001) (Table 2),

**Table 2:** Association of rs1799971 with psoriasis vulgaris analysed by subgroups (onset, familial vs. sporadic and severity)

Phenotype groups	Additive (GG vs. AA)	*p* value	Recessive (GG vs. GA + AA)	*p* value	Dominant (GG + GA vs. AA)	*p* value
	OR (95% CI)		OR (95% CI)		OR (95% CI)	
Early-onset psoriasis (≤ 40 years) vs. late -onset (> 40 years)	0.66 (0.44–1.01)	0.054	1.17 (0.17–7.85)	0.871	0.62 (0.39–0.97)	0.035
Familial psoriasis vs. sporadic psoriasis	**1.99 (1.36–2.91)**	**0.0003**	4.53 (0.77–26.4)	0.092	**2.01 (1.35–3.01)**	**0.0006**
Severe psoriasis (PASI ≥ 10) vs. mild psoriasis (PASI < 10)	0.97 (0.65–1.45)	0.900	0.79 (0.13–4.85)	0.808	1.05 (0.68–1.59)	0.829

Significant results for rs1799971 *(OPRM1)* were found when familial cases were compared to sporadic cases (highlighted in bold)

When creating an additional subgroup from psoriatic patients with history of early onset (≤ 40 years) and familial aggregation and comparing it to the HG population, in case of the rs1229984 (*ADH1B*) and rs1799971 (*OPRM1*) significant associations were found. The effect allele ‘C’ (rs1229984) both in the additive and recessive models, increased the risk of psoriasis similarly to the finding represented in Table 1. However, in the subgroup analysis the OR was much larger (OR_additive_ = 2.41, 95% CI 1.26–4.61, *p* < 0.01, OR_recessive_ = 2.42, 95% CI 1.26–4.68, *p* < 0.01, Table 3). Furthermore, the G allele of the rs1799971 (*OPRM1*) was also significantly associated with increased psoriasis risk in the additive and dominant models (OR_additive_ = 1.75, 95% CI 1.27–2.43, *p* = 0.001, OR_dominant_ = 1.82, 95% CI 1.26–2.63, *p* = 0.001) (Table 3).

**Table 3:** Associations of rs1799971 and rs1229984 in case of additional psoriasis vulgaris subgroups (history of early onset (≤ 40 years) and familial aggregation) compared to the general population

SNP	Effect alelle	Phenotype groups vs. HG	Additive (GG/CC vs. AA)	*p* value	Recessive (GG/CC vs. GA/CA + AA)	*p* value	Dominant (GG/CC + GA/CA vs. AA)	*p* value
			OR (95% CI)		OR (95% CI)		OR (95% CI)	
rs1799971	G	Early-onset (≤ 40 years) + familial aggregation vs. general population	**1.75 (1.27–2.43)**	**0.001**	2.57 (0.87–7.62)	0.088	**1.82 (1.26–2.63)**	**0.001**
rs1229984	C	Early-onset (≤ 40 years) + familial aggregation vs. general population	**2.41 (1.26–4.61)**	**0.008**	**2.42 (1.26–4.68)**	**0.008**	1.08 (0.1–2.3)	0.998

In case of rs1229984 (*ADH1B*) and rs1799971 (*OPRM1*) significant associations were found when the subgroup from psoriatic patients with history of early onset (≤ 40 years) and familial aggregation was compared to the HG population (highlighted in bold)

Familial aggregation and early onset of psoriasis inevitably suggest the involvement of genetic factors and is highly associated with HLA-Cw*0602. In our cohort, psoriatic patients who had a history of familial aggregation, the risk allele of HLA-Cw*0602 gene occurred more frequently compared to patients having sporadic disease (53.80% vs. 34.78%, *p* < 0.001, respectively), while among patients who had early-onset disease the proportion of those having at least one HLA-Cw*0602 risk allele was higher compared to those who had late onset disease (47.99% vs 28.08%, *p* < 0.001, respectively) (Supplementary Table 3). However, neither rs1229984 (ADH1B) nor rs1799971 (OPRM1) showed a significant association when subgroups formed on the presence of at least one HLA-Cw*0602 allele were compared. Assessing a possible gene–gene interaction between HLA-Cw*0602 and the ADH1B and OPRM1 genes in the early-onset vs. late-onset and familial aggregation vs. sporadic subgroups of patients no evidence on epistasis was found in any of the subgroups.

Furthermore, there were no differences in the risk allele distribution among HLA-Cw*0602 positive men and women in case of any SNPs.

## Discussion

The association between psoriasis and increased alcohol consumption has been reported in several studies, including the conclusion that alcohol consumption or abuse is an independent risk factor for the development of the disease. However, whether this association is driven by genetic factors has not been answered yet and therefore, in this study we evaluated the relationship between 23 SNPs related to increased alcohol intake and dependence in a Hungarian psoriasis group. We found that the frequency of the genetic variant rs1229984 (*ADH1B*) increased in the whole psoriasis group, while genetic variant rs1799971 (*OPRM1)* showed higher prevalence in the familial form of psoriasis patients. Importantly, the risk of psoriasis related to these variants increased further in the subgroup of psoriatic patients with history of early onset and familial aggregation, but with no association to the HLA-Cw*0602 allele.

Keeping the limitations in mind, it is intriguing to speculate on how the detected SNPs, which are primarily related to affecting the behaviour of an individual, may be involved also in the pathogenesis of psoriasis.

ADH1B is a key enzyme in the metabolism of ethanol to acetaldehyde and subsequent oxidation to acetate. The allele G which was increased in the psoriasis population leads to an increased enzyme activity, thus to decreased levels of the harmful acetaldehyde following alcohol ingestion. In contrast, the protective allele is linked to a high blood acetaldehyde concentration, which is accounted to make drinking unpleasant, and is behind the “Oriental flushing response” characterized by facial flushing, headache, tachycardia, and nausea [19, 30]. These symptoms altogether are considered to be a genetic deterrent to heavy drinking and alcoholism among East-Asians, where the predisposing alleles in rs1229984 of *ADH1B* is around 10% in contrast to the 90% found in the Hungarian and European populations [26]. Importantly, ADH1B is expressed not just in the liver where the majority of the alcohol metabolism takes place, but in the skin as well [5]. Moreover, its metabolite acetaldehyde, was found to induce the proliferation [14] and the production of pro-inflammatory cytokines in keratinocytes under in vitro conditions, that could provide a missing piece in the puzzle of how alcohol could increase the severity of psoriasis. Based on these data, further studies could reveal important keratinocyte specific differences in the allele carrying patients. Another interesting aspect for further investigations is that ADH1B is most likely capable of metabolizing retinoic acid as well which could account for an altered response of psoriatic patients to retinoic acid based therapies [29].

*OPRM1* encodes the *μ*-opioid receptor, which upon activation by its ligands, such as opioids and analgesic agents such as beta-endorphin, modulates the dopamine system [22]. It is implicated in complex behavior patterns such as alcohol dependence in Caucasian, native American tribes as well as in a study group of Asian descends [4, 8, 23, 24] in alcohol dependence associated impulsivity [35] just as in a reduced response to rewarding stimuli [25, 39]. Out of the alleles, 118G has the major susceptibility effect. In individuals carrying 118G stimulation, sedation, and positive mood levels after alcohol intake were significantly higher than in controls [39]. These effects are confirmed in great by studies on its antagonist, naltrexone, which is prescribed to alcohol dependent people to help them reduce cravings, control or abstain from drinking [28]. However, there are also studies that did not find a higher risk for alcohol dependence among *OPRM1* 118G-allele carriers, just as there are alcohol dependent patients who do not observe the beneficial effects of naltrexone [1, 16, 42]. Thus, patient characteristics and the genetic/environmental settings that modulate the *OPRM1* related behavior needs to be defined in more details. Regarding psoriasis, there is a significant amount of data on the impact of psoriasis on health behavior by causing psychological stress and psychosocial disability [15]. However, how neurotransmitters are involved in the observed changes and could integrate into the psoriasis skin-brain axis has been addressed only in limited details [21, 40]. Importantly, opioid receptors, besides the CNS, are also expressed in the epidermis (*μ* and *κ* isoforms) with a possible role in transmitting the sensation of itch. While activation of *μ*-opioid receptors induces pruritus, activation of *κ*-opioid receptors is suggested to have a suppressive effect [2]. Moreover, the *κ*-opioid, but not the *μ*-opioid receptor was down-regulated in psoriasis skin [47]. These results altogether put forward the question how itch is affecting the alcohol use of psoriasis patients and how the detected risk allele of *OPRM1* could contribute. Our results, therefore, open new perspectives to stratify psoriasis subgroups also based on itch and alcohol use [51]. Moreover, the association between psoriasis and the opiate signaling pathway, provides excellent start points for further studies on understanding not just psoriasis associated stress and related risk behavior but also to identify psychiatric disorders that may be linked to psoriasis or at least to subsets of psoriatic patients on the level of genetic association [41].

A limitation of this study is that although both SNPs were confirmed to be significant in our two independent Hungarian study subgroups, their prevalence did not reach significance values in a psoriasis cohort [33] from Sweden. Therefore, these findings call for further studies to map these SNPs in a geographic distribution. While, to draw conclusions on the functional relevance of the identified SNPs regarding the behavior, and perhaps the symptoms and the response to specific therapies such as topical *μ*-opioid receptor antagonists [45] in a subgroup of psoriatic patients with itch, a larger sample size is needed than what we used for this study.

In conclusion, our data suggests that genetically defined high-risk individuals for alcohol consumption are more common among psoriasis patients in the general population in Hungary, which calls for further studies on how genetic determinants of health behavior could be integrated into the psoriasis—psychic stress—alcohol consumption cycle, just as into psoriasis management.

## Electronic supplementary material

Below is the link to the electronic supplementary material.

Supplementary material 1 (DOCX 48 kb)

Supplementary material 2 (DOCX 17 kb)

Supplementary material 3 (DOCX 17 kb)
