# Supplementary material for: The prevalence of ADH1B and OPRM1 alleles predisposing for alcohol consumption are increased in the Hungarian psoriasis population
Source: Arch Dermatol Res. 2019 Apr 22;311(6):435–42. doi: 10.1007/s00403-019-01915-y (PMC6594982; doi:10.1007/s00403-019-01915-y)
Supplement: Supplementary file 1 — Supplementary material 1 (DOCX 48 kb) [file 403_2019_1915_MOESM1_ESM.docx]

**Supplementary Table 1.**

Selected SNPs and the effect of their minor alleles on alcohol consumption

| **Genes** | **SNPs** | **Described associations** | **Effect of minor allele** | **References** |  |
| --- | --- | --- | --- | --- | --- |
| ADH1C | rs1693482 | Alcohol dependence , alcoholism, maximum number of drinks per day | susceptible | 1 |  |
| ADH4 | rs7694646 | Drug dependence, cocaine dependence, alcohol dependence | susceptible | 2 |  |
| ADH5 | rs1154400 | Alcohol dependence | susceptible | 3 |  |
| ADH7 | rs1154458 | Alcoholism | susceptible | 4 |  |
| GABRA2 | rs279858 | Alcohol dependence | susceptible | 5 |  |
|  | rs567926 | Alcohol dependence | susceptible | 5, 6 |  |
| SLC6A3 | rs463379 | Harmful alcohol consumption | susceptible | 7 |  |
| DDC | rs3779084 | Alcohol consumption quantity | susceptible | 8 |  |
| OPRK1 | rs6985606 | Alcohol dependence | susceptible | 9 |  |
| OPRM1 | rs1799971 | Alcohol dependence | susceptible | 10 |  |
| POMC | rs1866146 | Alcohol dependence | susceptible | 11 |  |
|  | rs6713532 | Alcohol dependence | susceptible | 11 |  |
| ADH1B | rs1229984 | Alcoholism | protective | 12, 13 |  |
| ADH4 | rs1800759 | Alcohol dependence | protective | 14 |  |
| ALDH1A1 | rs610529 | Harmful alcohol consumption | protective | 15 |  |
| ALDH2 | rs671 | Alcoholism | protective | 16 |  |
| BDNF | rs6265 | Substance-related disorders | protective | 17 |  |
| GABRA2 | rs279871 | Alcohol dependence | protective | 5 |  |
| GABRG1 | rs2221020 | Alcoholism | protective | 18 |  |
| HTR1B | rs130058 | Alcohol dependence | protective | 19 | |
| SLC6A3 | rs6530 | Harmful alcohol consumption | protective | 7 | |
| TPH2 | rs1386496 | Alcohol consumption quantity | protective | 8 | |
| MAOA | rs979606 | Alcohol dependence | protective | 20 | |
| GRIN2A | rs2072450 | Alcohol dependence | protective | 21 | |
| CHRM2 | rs324650 | Alcohol dependence | protective | 22 | |

1. Macgregor S, Lind PA, Bucholz KK, et al. Associations of ADH and ALDH2 gene variation with self report alcohol reactions, consumption and dependence: an integrated analysis. Hum Mol Genet. 2009;**18**:580-93.

2. Edenberg HJ, Xuei X, Chen HJ, et al. Association of Alcohol Dehydrogenase Genes with Alcohol Dependence: a comprehensive analysis. Hum Mol Genet. 2006;**15**:1539-49.

3. Luo X, Kranzler HR, Zuo L, et al. Diplotype Trend Regression Analysis of the ADH Gene Cluster and the ALDH2 Gene: Multiple significant Associations with Alcohol Dependence. Am J Hum Genet. 2006;**78**:973-87.

4. Osier MV, Lu R-B, Paksits AJ, et al. Possible Epistatic Role of ADH7 in the Protection Against Alcoholism. American Journal of Medical Genetics Part B (Neuropsychiatric Genetics). 2004;**126B**:19-22.

5. Cui WY, Seneviratne C, Gu J, Li MD. Genetics of GABAergic signaling in nicotine and alcohol dependence. Hum Genet. 2012;**131**:843–55.

6. Zintzaras E. Gamma-aminobutyric acid A receptor, α-2 (GABRA2) variants as individual markers for alcoholism: a meta-analysis. Psychiatr Genet. 2012;**22**:186-96.

7. Lind PA, Eriksson CJ, Wilhelmsen KC. Association between harmful alcohol consumption and dopmine transporter (DAT1) gene polymorphisms in a male Finnish population. Psychiatr Genet. 2009;**19**:117-25.

8. Agrawal A, Lynskey MT, Todorov AA, et al. A candidate gene Association study of alcohol consumption in young women. Alcohol Clin Exp Res. 2011;**35**:550-8.

9. Xuei X, Dick D, Flury-Wetherill L, et al. Association of the k-opioid sytem with alcohol dependence. Mol Psychiatry. 2006;**11**:1016-24.

10. Chena D, Liua L, Xiaoa Y, et al. Ethnic-specific meta-analyses of association between the OPRM1 A118G polymorphism and alcohol dependence among Asians and Caucasians. Drug Alcohol Depen. 2012;**123**:1-6.

11. Zhang H, Kranzler HR, Weiss RD, et al. Pro-Opiomelanocortin Gene Variation Related to Alcohol or Drug Dependence: Evidence and Replications Across Family- and Population-based Studies. Biol Psychiatry. 2009;**66**:128–36.

12. Li D, Zhao H, Gelernter J. Strong Association of The Alcohol Dehydrogenase 1B Gene (ADH1B) With Alcohol Dependence And Alcohol-induced Medical Diseases. Biol Psychiatry. 2011;**70**:504-12.

13. Toth R, Fiatal S, Petrovski B, McKee M, Adany R. Combined effect of ADH1B RS1229984, RS2066702 and ADH1C RS1693482/ RS698 alleles on alcoholism and chronic liver diseases. Dis Markers 2011;**31**:267-77.

14. Guindalini C, Scivoletto S, Ferreira RGM, et al. Association of Genetic Variants in Alcohol Dehydrogenase 4 With Alcohol Dependence in Brazilian Patients. Am J Psychiatry. 2005;**162**:1005-7.

15. Lind PA, Eriksson CJ, Wilhelmsen KC. The role of aldehyde dehydrogenase-1 (ALDH1A1) polymorphisms in harmful alcohol consumption in a Finnish population. Hum Genomics. 2008;**3**:24-35.

16. Li D, Zhao H, Gelernter J. Strong Proective Effect of The Aldehyde Dehidrogenase Gene (ALDH2) 504lys (*2) Allele Against Alcoholism and Alcohol-Induced Medical Diseases in Asians. Hum Genet. 2012;**131**:725-37.

17. Gratacos M, Gonzalez JR, Mercader JM, de Cid R, Urretavizcaya M. Brain-Derived Neurotrophic Factor Val66Met and Pschiatric Disorders: Meta-Analysis of Case-Control Studies Confirm Association to Substance Realetd Disorders, Eating Disorders, and Schizophrenia. Biol Psychiatry. 2007;**61**:911-22.

18. Enoch MA, Hodgkinson CA, Yuan Q, Albaugh B, Virkkunen M. GABRG1 ad GABRA2 as Independent Predictors for ALcoholism in Two Populations. Neuropsychopharmacol. 2009;**34**:1245-54.

19. Cao JX, Hu J, Ye XM, et al. Association between the 5-HTR1B gene polymorphisms and alcohol dependence in a Han Chinese population. Brain Research. 2011;**1276**:1-9.

20. Wang KS, Liu X, Aragam N, et al. Family-based association analysis of alcohol dependence in the COGA sample and replication in the Australian twin-family study. J Neural Transm. 2011;**118**:1293-9.

21. Schumann G, Johann M, Frank J, et al. Systematic Analysis of Glutamatergic Neurotransmission Genes in Alcohol Dependence and Adolescent Risky Drinking Behavior. Arch Gen Psychiatry. 2008;**65**:826-38.

22. Wang JC, Hinrichs AL, Stock H, et al. Evidence of common and specific genetic effects: association of the muscarinic acetylcholine receptor M2 (CHRM2) gene with alcohol dependence and major depressive syndrome. Hum Mol Genet. 2004;**13**:1903-11.
