# Supplementary material for: The prevalence of ADH1B and OPRM1 alleles predisposing for alcohol consumption are increased in the Hungarian psoriasis population
Source: Arch Dermatol Res. 2019 Apr 22;311(6):435–42. doi: 10.1007/s00403-019-01915-y (PMC6594982; doi:10.1007/s00403-019-01915-y)
Supplement: Supplementary file 2 — Supplementary material 2 (DOCX 17 kb) [file 403_2019_1915_MOESM2_ESM.docx]

**Supplementary Table 2.**

|  | Age of onset | | Aggregation/Occurence | | PASI | |
| --- | --- | --- | --- | --- | --- | --- |
|  | Early-onset | Late-onset | Familial | Sporadic | <10 | ≥10 |
|  | N=425 | N=228 | N=171 | N=408 | N=342 | N=161 |
| Age (year) mean±SD p-value | 44.18±14.64 | 61.40±8.98 | 45.15±16.79 | 52.47±14.46 | 49.85±16.28 | 50.10±13.35 |
|  | **<0.001**^a^ | | **<0.001**^a^ | | 0.708^a^ | |
| Gender N (%) Man | 275 (64.71) | 132 (57.90) | 111 (64.91) | 246 (60.29) | 206 (60.23) | 102 (63.35) |
| Woman | 150 (35.29) | 96 (42.10) | 60 (35.09) | 162 (39.71) | 136 (39.77) | 59 (36.65) |
| p-value | 0.087^b^ | | 0.297^b^ | | 0.503^b^ | |
| Severity N (%) <10 | 206 (64.17) | 135 (75.00) | 91 (60.27) | 251 (71.31) | - | |
| ≥10 | 115 (35.83) | 45 (25.00) | 60 (39.73) | 101 (28.69) |  |  |
| p-value | **0.013**^b^ | | **0.015**^b^ | |  |  |
| a Mann-Whitney U test |  |  |  |  |  |  |
| b χ^2^ test |  |  |  |  |  |  |

The comparison of familial aggregation and sporadic occurrence between psoriatic patients did not show a significant difference in case of gender (p=0.297). The mean age of psoriatic patients with sporadic occurrence was 52.47 years±14.46 with familial aggregation (45.15 years±16.79) (p<0.001). In respect of PASI score significant difference was observed, the proportion of psoriatic patients with severe PASI was significantly higher among the familial aggregation patients than in the sporadic occurrence group (p=0.015).
