# Supplementary material for: The prevalence of ADH1B and OPRM1 alleles predisposing for alcohol consumption are increased in the Hungarian psoriasis population
Source: Arch Dermatol Res. 2019 Apr 22;311(6):435–42. doi: 10.1007/s00403-019-01915-y (PMC6594982; doi:10.1007/s00403-019-01915-y)
Supplement: Supplementary file 3 — Supplementary material 3 (DOCX 17 kb) [file 403_2019_1915_MOESM3_ESM.docx]

**Supplementary Table 3.**

1. **The allele distribution of the HLA-Cw*0602 gene polymorphism in psoriasis subgroups of early vs. late onset**

| HLA-Cw*0602 gene polymorphism | late onset | early onset |
| --- | --- | --- |
| 0 risk allele | 72.13% | 52.01% |
| 1 risk allele | 26.44% | 44.22% |
| 2 risk alleles | 1.44% | 3.78% |

Among the patients who had early-onset disease the proportion of those having at least one HLA-Cw*0602 risk allele was higher compared to those who had late onset disease (47.99% vs 28.08 %, p<0.001 respectively).

1. **The allele distribution of the HLA-Cw*0602 gene polymorphism in psoriasis subgroups of sporadic vs. familial aggregation**

| HLA-Cw*0602 gene polymorphism | sporadic | familial aggregation |
| --- | --- | --- |
| 0 risk allele | 65.33% | 46.20% |
| 1 risk allele | 32.50% | 49.12% |
| 2 risk alleles | 2.17% | 4.68% |

Among those psoriatic patients who had familial aggregation in their history the risk allele of HLA-Cw*0602 gene occurred more frequently compared to the patients having sporadic disease (53.80% vs. 34.78%, p<0.001 respectively).
